# HPTLC – Bioautographic methods for selective detection of the antioxidant and α-amylase inhibitory activity in plant extracts

**DOI:** 10.1016/j.mex.2018.07.013

**Published:** 2018-07-20

**Authors:** Snezana Agatonovic-Kustrin, David W. Morton

**Affiliations:** a School of Pharmacy, Monash University Malaysia, Jalan Lagoon Selatan, Bandar Sunway, 47500, Selangor Darul Ehsan, Malaysia; b School of Pharmacy and Applied Science, La Trobe Institute of Molecular Sciences, La Trobe University, Edwards Rd, Bendigo, 3550, Australia

**Keywords:** Measuring α-amylase inhibition, α-Amylase inhibition, Bioautography, Bioassay, Phytochemical analysis, Stigmasterol

## Abstract

A high-performance thin-layer chromatography (HPTLC) method was developed for quantification of α-amylase inhibitory activity and stigmasterol content in ant plant extracts. An improved HPTLC method for the determination of total free radical scavenging activity in samples using DPPH• is also reported. For quantification of *α*-amylase inhibitory activity, the developed HPTLC plate is dipped into an *α*-amylase solution, and the bioautogram is then incubated at 25 °C for 30 min under humid conditions. For visualization of enzyme inhibitory activity, the starch test with an iodine indicator solution is used. The blue zone observed comes from the starch-iodine complex formed from starch that was not hydrolyzed by the amylase due to enzyme inhibition by the compound(s) present in the sample. The area of the blue zones was used to compare and quantify relative α-amylase inhibitory activity in different extracts. Location of the blue zones (hRF) on the plate was used to detect compounds that are responsible for the α-amylase inhibitory activity. Relative α-amylase activity was not related to the antioxidant activity, but was highly correlated with the stigmasterol content in the sample extracts (*R* = 0.95). Therefore, plant sterols present in the extracts might be responsible for α-amylase inhibitory activities in the extracts.

• The developed method for quantification of α-amylase inhibitory activity provides an efficient and effective tool that can be used to screen, detect and quantify α-amylase inhibitory activity in plant extracts.
• The proposed protocol is easy to run, involves minimal sample preparation, with multiple samples able to be analyzed in parallel on the same chromatographic plate, in a short time.
• There were significant differences in *α*-amylase inhibitory activity, stigmasterol content, and total free radical scavenging activity between methanol, ethanol, dichloromethane, and ethyl acetate ant plant extracts.

## Graphical abstract

**Figure:**
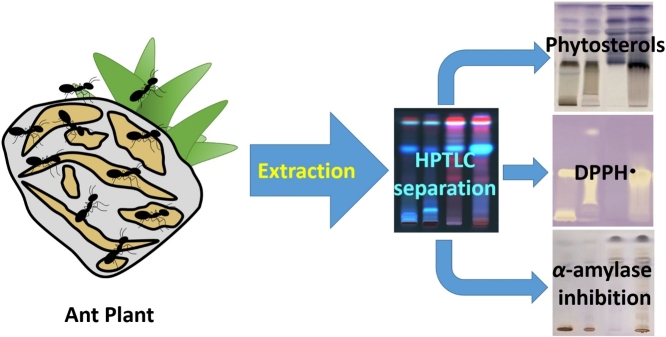

**Table:** 

Subject area	Chemistry
More specific subject area	Directed detection of bioactive compounds in complex samples.
Method name	Measuring α-amylase inhibition.
Name and reference of original method	High-performance thin-layer chromatographic methods in the evaluation of the antioxidant and anti-hyperglycemic activity of *Myrmecodia platytyrea*as a promising opportunity in diabetes treatment, J. Chromatogr. A, 1530 (2017) 192–196. https://doi.org/10.1016/j.chroma.2017.11.024.
Resource availability	N/A

## Method details

### Chemicals

2,2-Diphenyl-1-picrylhydrazyl free radical (DPPH•), gallic acid (97%), stigmasterol, and ethyl acetate were purchased from Sigma-Aldrich (Munich, Germany). Acetic acid, and methanol were purchased from Merck (Darmstadt, Germany), *n*-hexane from BDH (Poole, England), anisaldehyde from ACROS Organics (New Jersey, USA), and Milli-Q (Millipore) purified water was used. HPTLC separations were performed on 20 × 10 cm normal phase Silica gel 60 F_254_ HPTLC glass plates (Merck, Darmstadt, Germany).

### Sample preparation

*Myrmecodia platytyrea* subsp. *antoinii* (Becc.) Huxley & Jebb tubers (commonly referred to a ant plant) were collected from West Papua, Indonesia. Plant tubers were cut into small pieces, dried and ground into powder. Fat components were removed from 800 g of sample with *n*-hexane in a defatting step. The defatted plant material was sequentially extracted using solvents with increasing polarity (dichloromethane, ethyl acetate, ethanol, and finally methanol). The solvents from each extract were then removed using a rotary evaporator under reduced pressure at 40 °C. Final extracts for analysis were prepared as 1.0 mg/mL solutions. A 1.0 mg/mL standard solution of stigmasterol in absolute ethanol and a 0.4% w/v DPPH• solution in methanol were prepared. An anisaldehyde reagent solution was freshly prepared by combining 1 mL anisaldehyde with a refrigerated solution of glacial acetic acid/concentrated sulfuric acid in methanol in the ratio of 0.5:50:1. A 1% w/v amylase solution was prepared by diluting approximately 1.25 mL (1 g) of *α*-amylase from *Bacillus licheniformis* liquid (Cat. No. A4862, Sigma-Aldrich, Denmark) with distilled water to 100 mL. The enzyme stock solution was then refrigerated at 4 °C until required.

### High-performance thin-layer chromatography

High-performance thin-layer chromatography (HPTLC) plates were pre-washed before use with a blank run of methanol and activated by drying in an oven at 100 °C for 30 min. 20.0 μL volumes of dichloromethane, ethyl acetate, ethanol, and methanol sample extracts were sprayed with nitrogen onto plates as 8 mm bands using the Automatic TLC Sampler 4 (ATS 4, CAMAG, Muttenz, Switzerland), 8 mm from the lower edge, with 14 mm distance from each side, and a minimum distance of 2 mm between each tracks. A seven-point calibration was performed by applying 1.0, 2.0, 4.0, 6.0, 8.0, 10.0 and 12.0 μL of stigmasterol standard solution per band. HPTLC plates were developed in an Automated Multiple Development Chamber (AMD2, CAMAG). For the DPPH• assay, a two-step (40: 80) gradient elution method was used with 100% methanol over a 40 mm developing distance in the first step, and *n*-hexane, ethyl acetate, acetic acid (20:9:1) over a 80 mm developing distance in the second step. For the stigmasterol and *α*-amylase inhibitory activity, a single step elution using a *n*-hexane, ethyl acetate, acetic acid (20:9:1) mobile phase, over a 80 mm developing distance was sufficient [1].

Derivatization was achieved by dipping a HPTLC plate into the derivatizing agent for 1 s using the Chromatogram Immersion Device (CAMAG, Muttenz, Switzerland). Plates derivatized with DPPH• solution, were stored in the dark for 30 min and then photographed. Plates derivatized with anisaldehyde-sulfuric acid were heated at 110 °C for 10 min and then photographed. Images of the plates were captured with a TLC-visualizer (CAMAG, Muttenz, Switzerland) [1].

### Method validation for stigmastrol quantification

Stigmasterol was well resolved in sample extracts on the developed plates after derivatization with anisaldehyde/sulfuric acid. It was observed as a purple zone under visible light or as a peak in HPTLC densitometric chromatograms at hRF = 66. The methods for stigmasterol quantification by digital image analysis and TLC/HPTLC densitometry were validated for linearity, specificity, repeatability, limit of detection and limit of quantification (Table 1). Quantitative analysis of chromatogaphic plates was performed with digital image analysis software Sorbfil TLC Videodensitometer (Sorbpolymer, Krasnodar, Russia) and with CAMAG TLC/HPTLC scanner 4 controlled by winCATS 3.1 software (CAMAG, Muttenz, Switzerland). The plates were scanned in automatic mode at a wavelength of 550 nm, using a slit width of 0.3 mm and a slit length of 4.0 mm. The working range was assessed by plotting chromatographic peak areas against standard concentration (μg/band). Linear ranges were established using a least squared regression analysis. Specificity was assessed by the ability to separate samples. Repeatability was assessed by applying three repetitions of each standard at three concentrations within the calibration curve. Variance between repetitions was expressed as a relative standard deviation (%RSD). The sensitivity of the established method was estimated in terms of the limit of quantitation (LOQ) and limit of detection (LOD). LOQ and LOD were calculated using equations LOD = 3 × *Sd*/*B* and LOQ = 10 × *Sd*/*B*, where *Sd* is the standard deviation of the peak areas of the standards (*n* = 3), taken as a measure of noise, and *B* is the slope of the corresponding calibration curve.

**Table 1:** Statistical parameters for stigmasterol determinations after post-chromatographic derivatization with anisaldehyde/sulfuric acid, using digital image analysis and the densitometric method at 550 nm (*n* = 3).

Standard	Analysis/method	Equation of the line	Range (μg)	Applied/band (μg)	RSD (%)	LOD (μg)	LOQ (μg)
Stigmasterol	Digital image analysis	*y* = 27591*x* – 14174 *R*^2^ = 0.98	1.0–12.0	1.0 4.0 8.0	13.94^a^ 3.64 5.44	0.4	1.2
Stigmasterol	Densitometric method	*y* = 391.5*x* + 121.9 *R*² = 0.98	1.0–12.0	1.0 4.0 8.0	13.37^a^ 3.53 4.53	0.4	1.4

a Below limit of quantification; *y* = Zone area (pixels); *x* = Applied amounts (μg); *R*^2^ = Coefficient of determination.

### Antioxidant activity

The antioxidant activity of *M. platytyrea* extracts was assessed with a direct HPTLC-DPPH• assay coupled with digital image analysis. Plates were developed with a two-step (40: 80) gradient elution method. Methanol was used in the first step to spread/stretch the bands of the polar compounds present in the sample and move them from the start. In this instance, this spreading out of the zones of more polar compounds, mostly polyphenolics with free radical scavenging activity, is desirable. By stretching out the yellow zones, saturation of the zone on the plate is avoided and better (more precise) quantification of the total free radical scavenging activity in the reaction with DPPH• in the sample is achieved. The second elution step with the less polar mobile phase (*n*-hexane, ethyl acetate, acetic acid (20:9:1)) was used to better separate the less polar compounds. Antioxidant activities, in terms of free radical scavenging activity of the separated compounds that are present in the samples, appeared as yellowish zones against the purple background on the plate. Total antioxidant activity was expressed as the sum or total area of the yellow zones for each sample extract (Table 2).

**Table 2:** Comparison of total area size (in pixels) for the antioxidant activity (yellow zones after derivatization with DPPH•), area size of zones corresponding to stigmasterol in the extracts, and total area size of blue zones for *α*-amylase inhibitory activity from digital plate image analysis.

Extract	Yellow zones with DPPH•	Stigmasterol zones	Blue zones for α-amylase inhibitory activity
Methanol	568957	134004	140608
Ethanol	679342	156316	155562
Dichloromethane	47902	317493	284084
Ethyl acetate	765880	268852	285061
Correlation (*R*) with α-amylase activity	0.37	0.97	

*R* = correlation coefficient.

### α-Amylase inhibitory activity assay

**Fig. 1:**
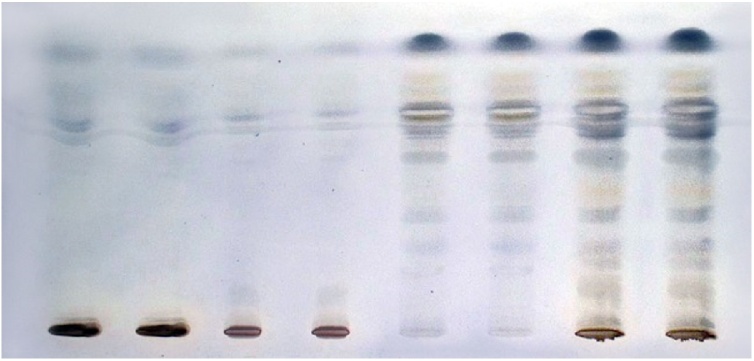
Starch-iodine method for α-amylase inhibitory activity. Tracks 1, 2 methanol extracts; Tracks 3, 4 ethanol extracts; Tracks 5, 6 dichloromethane extracts; Tracks 7, 8 ethyl acetate extracts.

*α*-Amylase inhibitory activity was estimated using the starch test with iodine solution as an indicator [2]. The developed plate was saturated with enzyme solution, and then incubated for 30 min at 37 °C in a humid chamber, in order for the primary reaction between the enzyme and any inhibitors present in a sample to react. After incubation, the plate was dipped in a 1% w/v starch solution, incubated for 10–20 min for enzyme substrate reaction, washed with Gram’s Iodine solution (detection solution), and then photographed. Starch produces a dark blue color on the HPTLC plate in the presence of iodine. A blue area around the zones indicates reduced or *α*-amylase inhibitory activity in the sample (Fig. 1). Therefore, plant extracts, positive for α-amylase inhibitors have blue zones after iodine staining at the positions of separated zones on the HPTLC plate, indicating the separated zone that is responsible for the inhibitory activity in the corresponding extract. The blue zone comes from the starch-iodine complex formed from starch that was not hydrolyzed by the amylase due to enzyme inhibition by the compound(s) in the extract sample. Ethanol and ethyl acetate extracts were found to have higher antioxidant activities due to the presence of polyphenolic antioxidants that are highly soluble in alcohol (Table 2). The dichloromethane extract does not have significant antioxidant activity, but shows significant *α*-amylase inhibitory activity. The ethyl acetate extract has significant antioxidant and anti-hyperglycemic activities.

Relative *α*-amylase inhibitory activity was also determined densitometrically by comparing the area size of the peaks in densitometric chromatograms obtained at 550 nm that correspond to the blue zones. The sum of the peak area size of the blue zones in the methanol extract was used as a standard reference to compare the total area size of blue zones in the remaining extracts (*i.e.* relative *α*-amylase inhibitory activity) (Table 3). Relative *α*-amylase inhibitory activity was highly correlated with the stigmasterol concentration in the sample extracts (*R* = 0.95).

**Table 3:** Stigmasterol content and relative α-amylase inhibitory activity estimated with HPTLC densitometric method at 550 nm.

Extract	Stigmasterol		*α*-amylase inhibitory activity	
	Peak area	Found (μg/20 μL)	Peak area (blue zones)	Relative inhibitory activity^a^
Methanol	1005	2.3	425	1.0
Ethanol	1113	2.6	1047	2.5
Dichloromethane	3149	7.7	19144	45.0
Ethyl acetate	3010	7.4	27954	65.7
		Correlation (*R*)	0.95	0.9

a Inhibitory activity in the methanol extract is used as a standard reference unit to express the relative α-amylase inhibitory activity in other extracts; *R* = correlation coefficient.

The HPTLC method for *α*-amylase inhibitory activity, based on the blue starch-iodine complex reaction to visualize the hydrolysis of starch by enzyme that is pre-absorbed on the developed plate, is simple, rapid, and does not require special equipment or instrumentation. It is highly suitable for high throughput screening of plant samples for the presence of components that inhibit *α*-amylase activity.

## Additional information

The management of diabetes and its related complications is a global problem. One of the therapeutic approaches for treating diabetes is the inhibition of *α*-amylase, an exoenzyme that hydrolyses starch to release glucose [3]. Since diabetic complications results from the oxidative stress and formation of hyperglycemia-derived oxygen free radicals that lead to oxidative degradation of glycated proteins [4,5], an antioxidant therapy combined with hypoglycemic drugs is recommended in order to avoid complications. The use of general antioxidants, like Vitamins C and E, has failed to demonstrate any beneficial effect in clinical trials [6]. Instead, the use of drugs with modest antioxidant activity has been shown to improve many of the problems associated with diabetic complications [7]. *Myrmecodia,* or ant plant, is a tropical woody plant widely distributed in equatorial region of the world, traditionally used in Irian Jaya as an alternative treatment for diabetes, as the substances produced by ants can reduce blood sugar levels. Strong antioxidant and antimicrobial activities have been observed in bioactive flavonoids and phenolic compounds isolated from the crude ethyl acetate extract from a related species *Hydnophytum formicarum* Jack. Stigmasterol was isolated from the crude hexane and dichloromethane extracts of this plant [8]. The antibacterial activities of stigmasterol and β-sitosterol [[9], [10], [11]], and the potent antioxidant, hypoglycemic, and thyroid inhibiting properties of stigmasterol have been previously reported [12,13].
